# miR-29a Downregulates PIK3CA Expression and Inhibits Cervical Cancer Cell Dynamics: A Comparative Clinical Analysis

**DOI:** 10.3390/cimb46110754

**Published:** 2024-11-08

**Authors:** Hyorim Jeong, Kangchan Choi, Dasom Hwang, Sunyoung Park, Yong Serk Park, Hyeyoung Lee

**Affiliations:** 1 College of Engineering and Medicine, University of Illinois Chicago, Chicago, IL 60612, USA; 2 School of Medicine, Trinity Medical Sciences University, Ribishi VC0272, Saint Vincent and the Grenadines; 3 Department of Biomedical Laboratory Science, Yonsei University, Wonju 26493, Republic of Korea; hdasom208@naver.com; 4 School of Mechanical Engineering, Yonsei University, Seoul 03722, Republic of Korea; sun-young@yonsei.ac.kr

**Keywords:** miRNA, miR-29a, PIK3CA, cervical cancer

## Abstract

HPV/pap tests are widely used for cervical cancer screening, playing a crucial role in early diagnosis and guiding future treatment options. However, approximately 50% of cervical cancer patients are diagnosed at an advanced stage, which is associated with higher recurrence rates and poorer survival outcomes than early-stage diagnoses. This underscores the need for effective treatments for advanced-stage cervical cancer. Among the various oncogenes implicated in cancer, PIK3CA expression is known to cause cervical cancer, suggesting that inhibiting PIK3CA may impede cervical cancer progression. In this study, we transfected PIK3CA-overexpressing tumor cells (SiHa, C33A, and HeLa) with miR-29a, a microRNA extensively studied as a therapeutic candidate for oncogene suppression in various tumor types. We conducted RT-qPCR and Western blot analyses to assess changes in PIK3CA expression at the RNA and protein levels. Wound healing and cell migration assays were used to evaluate the effects of miR-29a on cell division and migration in HeLa cells. We confirmed a reduction in PIK3CA expression at both RNA and protein levels following miR-29a transfection. After transfecting miR-29a into HeLa cells, we observed a reduction in cell division and migration, as demonstrated by wound healing and cell migration assays. Additionally, we found that miR-29a binds to the 3′-UTR region of PIK3CA, leading to a reduction in its gene expression. Furthermore, we correlated the concentration of miR-29a in clinical histologic biopsy samples from cervical cancer patients with disease progression. These findings indicate that miR-29a can slow the progression of cervical cancer by targeting PIK3CA and potentially aid in its treatment. miR-29a shows promise as a therapeutic agent for inhibiting oncogene expression and controlling cervical cancer progression, especially in advanced-stage cases.

## 1. Introduction

Cervical cancer is the fourth most common cancer among women worldwide. In 2022, approximately 20 million new cases and 9.7 million deaths were reported globally [1]. The primary cause of cervical cancer is persistent infection with high-risk human papillomavirus (HR-HPV) [2,3,4]. Screening methods such as the Papanicolaou test and selective HR-HPV testing are effective for diagnosing and treating cervical cancer at pre-cancerous stages [5]. However, despite these preventive measures, approximately 50% of cervical cancer patients are diagnosed at an advanced stage, according to the International Federation of Gynecology and Obstetrics (FIGO) [6,7,8]. These advanced-stage cases are not amenable to surgical treatment, and the standard treatment of chemoradiotherapy often results in high recurrence rates with limited survival benefits [9,10]. Consequently, there is a pressing need for new treatments for advanced cervical cancer.

Oncogene-targeted therapies have garnered significant attention in recent years. Several FDA-approved drugs, such as trastuzumab (Herceptin) for Human Epidermal growth factor Receptor 2 (HER-2) positive breast and stomach cancers and vemurafenib (Zelboraf) for BRAF-mutant metastatic melanoma, exemplify the success of this approach [11,12]. In cervical cancer, the phosphatidylinositol 3-kinase catalytic subunit alpha (PIK3CA) gene is a critical oncogene. Mutations and amplifications in PIK3CA lead to the activation of the AKT signaling pathway, promoting abnormal cell survival and proliferation [13,14,15]. The Cancer Genome Atlas (TCGA) database indicates high frequencies of PIK3CA mutations and copy number amplifications in cervical cancer patients, making PIK3CA a promising therapeutic target. Preclinical studies have shown that PI3K inhibitors can reduce the oncogenic activity of cervical cancer cells [16,17]. As such, it has been well documented that PIK3CA is overexpressed in cervical cancer. Our study aims to directly target the expression of PIK3CA to treat cervical cancer and inhibit tumor progression.

MicroRNAs (miRNAs) have emerged as potent tools for oncogene suppression. These small non-coding RNAs, consisting of 18–25 nucleotides, form RNA-induced silencing complexes that bind the 3′-untranslated region (UTR) of target mRNAs. This binding can result in either mRNA degradation (if the binding is perfect) or translational repression (if the binding is imperfect) [18,19]. The fundamental role of miRNAs is to regulate protein translation, making them versatile tools for gene expression modulation. Small interfering RNAs (siRNAs) also silence genes, but they differ from miRNAs in several key aspects [20,21]. While siRNAs target a single gene, miRNAs can regulate multiple genes simultaneously. Structurally, miRNAs possess a hairpin loop, which enhances their stability compared to siRNAs. Moreover, miRNAs are naturally occurring regulators in the body, potentially offering a safer therapeutic profile [22].

miRNAs can either promote or inhibit tumor formation by regulating oncogenes or tumor-suppressor genes. Several tumor-suppressor miRNAs are currently under investigation for cancer treatment. For instance, microRNA-34a acts as a tumor suppressor in various solid cancers, and clinical trials are underway for miRNA-based treatments [23,24,25,26]. Among these, miR-29a has demonstrated tumor-suppressor functions across multiple cancer types, including cervical, gastric, pancreatic, prostate, head and neck, colorectal, and glioma [27,28]. Studies have shown that miR-29a targets several mRNAs in cervical cancer, such as NAD-dependent deacetylase sirtuin-1 (SIRT1), cell division regulatory protein 42 isoform (CDC42), and suppressor of cytokine signaling-1 (SOCS1) [29,30].

This study aims to investigate the effects of miR-29a on cervical cancer cell survival, migration, and proliferation, specifically focusing on its ability to inhibit PIK3CA. Given that PIK3CA is well known to be overexpressed in cervical cancer and that miR-29a has demonstrated growth inhibitory effects in various tumors by modulating the PI3K/AKT/mTOR signaling pathway, we conducted this study to determine whether miR-29a directly affects PIK3CA. Additionally, we compared miR-29a levels in the blood of cervical cancer patients with those of healthy individuals to further elucidate its role in cervical cancer development. By targeting PIK3CA using miR-29a, we hope to offer a novel therapeutic strategy for treating advanced cervical cancer.

## 2. Materials and Methods

### 2.1. Cell Lines and Cell Culture

Human cervical cancer cell lines (SiHa, ATCC HTB-35; HeLa, ATCC CCL-2; C33A, ATCC HTB-31) were purchased from the American Type Culture Collection (ATCC, Manassas, VA, USA). SiHa, HeLa, and C33A cells were cultured in Dulbecco’s Modified Eagle’s Medium (DMEM; Gibco, Carlsbad, CA, USA) supplemented with 10% fetal bovine serum (FBS; Gibco) and 1% streptomycin–penicillin (Gibco). All cell lines were incubated at 37 °C in a humidified 5% CO_2_ incubator. The cells were subcultured at 80–90% confluence.

### 2.2. Total RNA Isolation

Total RNA was isolated from cervical biopsy tissue, tumor tissue from patients, and normal individuals, as well as from cervical cancer cell lines, using the RNeasy Mini Kit (Qiagen, Hilden, Germany). Briefly, the formalin-fixed paraffin-embedded (FFPE) RNA expression was analyzed for tissue samples. Five tissue sections, each 6 μm thick, were mixed with 1 mL of xylene and 10 IU of Proteinase K, followed by an overnight incubation at 37 °C. RNA extraction was then carried out using the same method described below for the cell lines. For the cell lines, the supernatant of the cell culture was removed, and the cells in a 6-well culture plate were washed three times with Phosphate-Buffered Saline (PBS; Gibco). After removing the PBS, 600 μL of lysis buffer RLT supplemented with 6 μL of β-mercaptoethanol was added to the cells and mixed thoroughly by pipetting. The cell lysate was then transferred to a 1.5 mL microcentrifuge tube, and 600 μL of 70% ethanol was added and mixed. Subsequently, 600 μL of the lysate was loaded into an RNeasy mini column in a 2 mL collection tube and centrifuged for 15 s at 8000× *g*. The flow-through was discarded, and an additional 600 μL of the lysate was loaded into the column and centrifuged again for 15 s at 8000× *g*. Next, 700 μL of buffer RW1 was added to the column and centrifuged at 8000× *g* for 15 s. The flow-through was discarded, and 500 μL of buffer RPE was added to the column and centrifuged for 15 s at 8000× *g*. Another 500 μL of buffer RPE was added to the column and centrifuged at 8000× *g* for 2 min. The RNeasy mini column was then transferred to a new collection tube and centrifuged for 1 min at full speed. The column was then placed in a new 1.5 mL microcentrifuge tube, and 50 μL of DEPC-treated water was added to the column and centrifuged at 10,000× *g* for 1 min to obtain total RNA. The purity and concentration of the total RNA were measured by the absorbance ratio at 260 and 280 nm using the Infinite 200 spectrophotometer (Tecan, Mannedorf, Switzerland). All procedures for handling the total RNA were carried out in a laminar flow hood under RNase-free conditions. The isolated total RNA was stored at –80 °C until use. The RNA was treated with the Turbo DNase kit (Life Technologies, Carlsbad, CA, USA) to digest dsDNA templates and was used for cDNA synthesis. Briefly, 1.5 μL of DEPC, 2.5 μL of TURBO DNase buffer, and 1 μL of TURBO DNase were added to 20 μL of the total RNA sample. After incubation at 37 °C for 30 min, 3 μL of DNase inhibitor was added and mixed by gentle tapping for 5 min in a flow hood. The reagents were then briefly centrifuged at full speed, and the supernatant was transferred to a 1.5 mL microcentrifuge tube.

### 2.3. Complementary DNA Synthesis

For cDNA synthesis using miRNA, complementary DNA (cDNA), reverse transcription was performed using a High Capacity cDNA Reverse Transcription Kit (Applied Biosystems, Foster City, CA, USA). Briefly, 2 ng/μL of RNA was used for cDNA synthesis. The reverse transcriptase (RT) mixture contained 0.15 μL of 100 mM dNTP mix (100 mM each of dATP, dGTP, dCTP, and dTTP at a neutral pH), 0.25 μL of 200 U/μL Murine Molony Leukemia Virus Reverse Transcriptase (MMLV-RT; Invitrogen, Carlsbad, CA, USA), 1.5 μL of 10× reverse transcriptase buffer, 0.19 μL of 20 U/μL RNase inhibitor, and 3 μL of 5× miRNA-specific primer, adjusted to a final volume of 15 μL with nuclease-free water. The following TaqMan small RNA assay (Applied Biosystems) primers were used: RNU6B and hsa-miRNA-29a. The cDNA synthesis was performed at 16 °C for 30 min, 42 °C for 30 min, and 85 °C for 5 min. For cDNA synthesis using mRNA, the cDNA was synthesized using the PrimeScript™ RT Master Mix (PrimeScript™ RTase, RNase Inhibitor, random 6-mers, Oligo dT Primer, dNTP mixture, and buffer; Takara, Shiga, Japan). Briefly, 10 μL of PrimeScript RT Master Mix, RNase-free water, and 2.5 ug of total RNA were mixed. The 50 μL PCR reaction was incubated at 37 °C for 60 min, 85 °C for 5 s, and then held at 4 °C.

### 2.4. RT-qPCR (Reverse Transcriptase Quantitative PCR) for Relative Gene Expression Analysis

The microRNA expression was quantified by determining the cycle threshold (Ct), which is the number of PCR cycles required for the fluorescence to exceed a value significantly higher than the background fluorescence using the TaqMan small RNA assays (Applied Biosystems) with miRNA-specific primers. Briefly, 2 μL of cDNA was added to 10 μL of 2× Thunderbird probe qPCR mix (Toyobo, Osaka, Japan), with 1 μL of 20× miRNA-specific primer and 7 μL of nuclease-free water, making a final volume of 20 μL. RT-qPCR reactions were performed on a CFX96 Real-Time PCR System Detector (Bio-Rad, Hercules, CA, USA). The samples were run in duplicate for each experiment. The RT-qPCR data for miR-29a were calculated by the comparative Delta Ct method (2^−ΔCt^) using RNU6B as the endogenous control. The relative expression level of PIK3CA was quantified similarly, but GAPDH was used as the endogenous control. Other conditions of the PCR cycle and the materials used for analyzing the expression of PIK3CA were the same as those used for miR-29a. To monitor reagent contamination, a negative control was included for each primer pair. PCR cycling conditions were 95 °C for 10 min, followed by 40 cycles of 95 °C for 15 s and 60 °C for 60 s.

### 2.5. Cell Migration Assay

Cell migration assays were performed using SPLScar™ (SPL, Pocheon, Republic of Korea). HeLa cells were seeded at a density of 4 × 10^5^ cells/well in 6-well culture plates. After 24 h, when the cells reached 70–80% confluence, they were transfected with miRNA-29a mimics and scrambled miRNAs. Following a 24 h incubation at 37 °C, the cells were washed three times with PBS, and monolayer cells were scratched to create 500 µm wide wound gaps using SPLScar™. The scratched cells were then cultured in DMEM supplemented with 10% FBS. The wound gaps were photographed using a light microscope (Olympus, Tokyo, Japan) at 400× magnification at 0, 24, 48, and 72 h after scratching. The percentage of migrated cells was analyzed and graphed using ImageJ software (version 1.53k; Media Cybernetics, Rockville, MD, USA).

### 2.6. Cell Invasion Assay

Cell invasion assays were performed using SPL Insert™ Hanging (SPL, Pocheon, Republic of Korea). The HeLa cells were transfected with miRNA-29a mimics and scrambled miRNA at a density of 4 × 10^5^ cells/well for 24 h. The transfected cell pellets were then resuspended in a serum-free culture medium, and 1 × 10^5^ HeLa cells/well were counted for subsequent seeding into the transwell. To create a basement membrane environment for cell invasion, the upper chamber of the transwell inserts was pre-coated with Matrigel Matrix (50 µL/cm^2^ growth area, BD Biosciences, Sparks, MD, USA). Next, 1 × 10^5^ resuspended cells were seeded into the upper chamber of the transwell inserts. In the lower chamber, 1 mL of culture medium containing 10% FBS was added to serve as a chemoattractant, encouraging the cells to invade and move through the transwell. The cells and transwell were incubated for 24 h. After incubation, the cells on the upper surface of the transwell insert were washed three times with cold PBS and removed using cotton swabs. The invaded cells on the lower surface of the transwell membrane were fixed with 1 mL of 4% paraformaldehyde for 30 min, then washed three times with 1 mL of cold PBS and stained with 0.05% crystal violet for one hour. After washing the transwell with PBS, the invaded cells in three random fields were observed using a light microscope and counted using ImageJ software. Three fields were randomly selected from each membrane, and the number of invaded cells was counted.

### 2.7. Dual-Luciferase Assay

To quantify the binding affinity between miRNA-29a and PIK3CA, the Dual-Luciferase Reporter Assay System (Promega, San Luis Obispo, CA, USA) was employed. Using the predicted 3′ UTR binding site of PIK3CA mRNA (Figure S2), the binding region of the wild-type or mutant-type was inserted into the regulatory site of the pmiRGLO Dual-Luciferase miRNA Target Expression Vector (pmiRGLO; Promega) via the Bioneer cloning service system (Bioneer, Daejeon, Republic of Korea). The sequences were as follows: PIK3CA wild-type: 5′-GTTTAAACAATATGTGGTGTTAATAGATTGUGGUGCUTTTACTATTTA AAGACAACTTTCATCTAGA-3′, PIK3CA mutant-type: 5′-GTTTAAACAATAT GTGGTGTTAATAGATTGACCACGATTTACTATTTAAAGACAACTTTCAT CTAGA-3′. The wild-type and mutant-type plasmids were co-transfected into HeLa cells at a density of 4 × 10^5^ cells/well with 200 pmole/well of miRNA-29a or scramble miRNA, respectively. After 24 h of transfection, the luciferase activity of the co-transfected HeLa cells was measured using the Dual-Luciferase Reporter Assay System (Promega) and a microplate luminometer (Centro XS3 LB960; Berthold, Oak Ridge, TN, USA). The relative luciferase activity was calculated as the ratio of firefly luciferase activity to Renilla luciferase activity.

### 2.8. Western Blotting Analysis

The HeLa cells were seeded at a density of 4 × 10^5^ cells/well in 6-well culture plates. After transfection with miRNA-29a mimics and scramble miRNA for 24 h, the cells were washed twice with PBS and then lysed in 50 μL of cold RIPA lysis buffer (RIPA; Boster Biological Technology, Pleasanton, CA, USA) and protease inhibitor cocktail (Roche Diagnostics, Indianapolis, IN, USA). The cell lysates were collected using a cell scraper (SPL), pooled in a 1.5 mL microcentrifuge tube, and centrifuged at 14,000 rpm at 4 °C for 30 min. The supernatants from the centrifuged cell lysates were transferred to a new 1.5 mL tube and used as protein samples. The total protein samples were quantified using the BCA Assay Kit (Thermo Fisher Scientific, Waltham, MA, USA). Ten micrograms of each protein sample were heated at 90 °C for 10 min with 5× reducing dye. The heated protein samples were run on SDS-PAGE in a 10% running gel at 100 V for 90 min and then transferred to nitrocellulose membranes (Bio-Rad) for 1 h at 350 milliamperes (mA). The membranes were immersed in 5% skim milk with TBST for 2 h to block nonspecific binding sites. The membrane was then incubated with primary antibodies against PIK3CA (1:1000, Rabbit mAb; Cell Signaling, Cat# 4249, Beverly, MA, USA) and GAPDH (1:1000, Mouse mAb; Cell Signaling, Cat# 51332) at 4 °C overnight. The next day, the membranes were washed three times with TBST for 10 min each and incubated with horseradish peroxidase-conjugated anti-mouse or anti-rabbit secondary antibodies (1:1000; Cell Signaling Technology, Danvers, MA, USA) for 2 h at room temperature. Protein bands were visualized using SuperSignal^®^ WestPico Plus (Thermo Fisher Scientific, Waltham, MA, USA). The band intensity was measured with a chemiluminescence imaging system (FUSION Solo; Vilber-Lourmat, Paris, France), and the relative protein expression was calculated using ImageJ software.

### 2.9. Statistical Analysis

Statistical analysis was performed using GraphPad Prism software version 9.0 (GraphPad Software, La Jolla, CA, USA). All experiments were repeated at least three times, and one representative result was presented. The Student’s *t*-test (two-tailed) was used to compare the control and experimental groups. The Kruskal–Wallis test was employed for the miR-29a expression level in varied cervical cancer cell lines and patient FFPE samples to determine the statistical significance as a non-parametric test. Additionally, *p*-values less than 0.05 were considered statistically significant.

## 3. Results and Discussion

### 3.1. Expression Analyses of miR-29a and PIK3CA in Cervical Cancer Cell Lines with Different HPV Statuses

**Figure 1:**
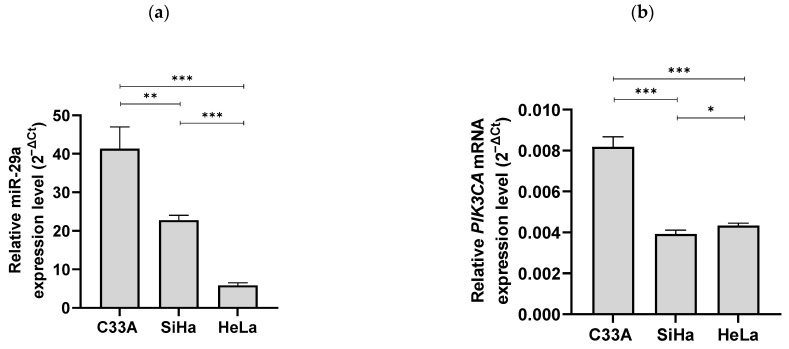
Differential expression of miR-29a and PIK3CA in HPV-negative and HPV-positive cervical cancer cell lines. (**a**) Relative expression levels of miR-29a in three cervical cancer cell lines: C33A (HPV-negative), SiHa (HPV type 16 positive), and HeLa (HPV type 18 positive), as determined by RT-qPCR. C33A cells exhibit the highest expression of miR-29a, whereas HeLa cells show the lowest levels. Error bars represent the standard deviation from three independent experiments. (**b**) Relative expression levels of PIK3CA in the same cell lines, as quantified by RT-qPCR. C33A cells show the highest PIK3CA expression, with both SiHa and HeLa displaying lower but comparable expression levels. Statistical significance is denoted as * *p* < 0.05; ** *p* < 0.01; *** *p* < 0.001.

The relative expression of miR-29a was quantified by RT-qPCR in three cervical cancer cell lines: C33A (HPV-negative), SiHa (HPV type 16 positive), and HeLa (HPV type 18 positive). The primers for the PIK3CA detection used in RT-qPCR were selected through multiple alignments (Figure S1 and Table S1). The C33A cells exhibited the highest expression of miR-29a, with a relative expression level of approximately 41.3. In contrast, the SiHa and HeLa cells, which represent HPV-infected cell lines, displayed significantly lower miR-29a expression levels of about 22.8 and 5.9, respectively. Specifically, the HeLa cells infected with HPV type 18 expressed only about one-eighth of the miR-29a levels observed in the C33A cells. This finding confirms that HPV-infected cervical cancer cell lines have markedly reduced miR-29a expression compared to HPV-negative cervical cancer cell lines (Figure 1a).

Using the same RT-qPCR method, we quantified the relative expression of PIK3CA in each cell line. Contrary to our expectations, the C33A cells exhibited the highest PIK3CA expression, with a relative expression level of approximately 0.0082 (normalized to GAPDH). The SiHa and HeLa cells showed similar but lower levels of PIK3CA expression, about 0.0039 and 0.0043, respectively. This observation suggests that PIK3CA overexpression is not necessarily correlated with HPV status in these cervical cancer cell lines (Figure 1b).

Given these results, we selected the HeLa cells for further study due to their high PIK3CA expression and notably low miR-29a levels. This choice allows us to investigate the potential therapeutic effects of miR-29a in a context where its expression is minimal, and PIK3CA is sufficiently expressed to observe meaningful changes upon treatment.

### 3.2. Effectiveness of miR-29a in Reducing PIK3CA Levels in HPV-Positive Cervical Cancer Cells

**Figure 2:**
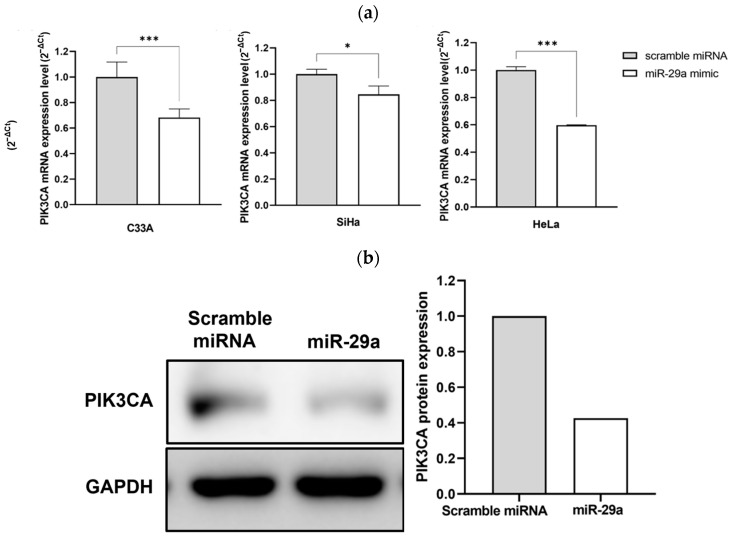
Inhibition of PIK3CA expression by miR-29a in cervical cancer cell lines. (**a**) Relative expression levels of PIK3CA mRNA in C33A (HPV-negative), SiHa (HPV type 16 positive), and HeLa (HPV type 18 positive) cervical cancer cell lines following transfection with 200 pmole of miR-29a, as determined by RT-qPCR. (**b**) Western blot analysis showing PIK3CA protein levels in HeLa cells transfected with either scramble miRNA (control) or miR-29a. Error bars represent the standard deviation from three independent experiments. Statistical significance is indicated as * *p* < 0.05; *** *p* < 0.001.

To determine whether miR-29a can inhibit PIK3CA in HPV-positive cervical cancer cell lines, we transfected each cell line with 200 pmole of miR-29a and calculated the relative expression level of PIK3CA at the RNA level. The results showed a significant reduction in PIK3CA expression in all cell lines, including C33A. Notably, among the HPV-positive cell lines, the HeLa cells exhibited a marked decrease in PIK3CA expression (Figure 2a). This reduction in PIK3CA RNA levels in the cervical cancer cell lines transfected with miR-29a indicates that miR-29a effectively induces gene silencing of PIK3CA. After confirming that miR-29a reduced PIK3CA expression at the RNA level, we performed Western blotting to determine if this reduction also occurred at the protein level. Protein expression was visualized using ImageJ (Figure 2b). In this experiment, scramble miRNAs, which are designed not to silence any genes, were used as the controls. The results showed that transfection with miR-29a reduced the protein expression of PIK3CA compared to the scrambled miRNA in HeLa cells. As previously mentioned, the HeLa cells exhibited the lowest expression of miR-29a and a comparable level of PIK3CA expression to other cervical cancer cells. Therefore, we conducted further experiments on the inhibition of tumor cell division and invasion by miR-29a, specifically in the HeLa cells.

### 3.3. Reduced Proliferation and Migration of HeLa Cells Induced by miR-29a Treatment

**Figure 3:**
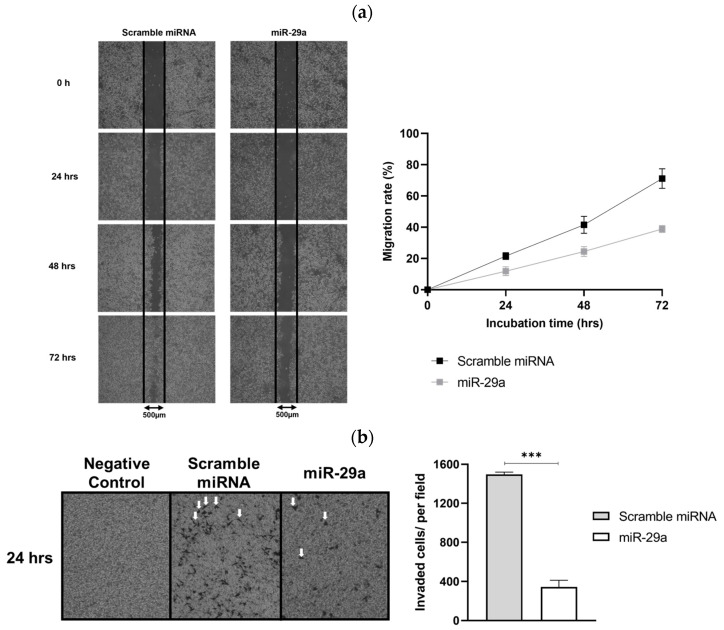
Effect of miR-29a on HeLa cell proliferation and migration. (**a**) Wound healing assay results for HeLa cells treated with scramble miRNA (control) and miR-29a demonstrate that miR-29a significantly inhibits cell growth and division. Images were taken at the indicated time points to assess the closure of the wound area. (**b**) Cell migration assay results for HeLa cells treated with scramble miRNA and miR-29a. The black specks (highlighted by white arrows) indicate the migrated cells. Statistical significance is denoted as *** *p* < 0.001. Error bars represent the standard deviation from three independent experiments.

HeLa cells are known to be highly proliferative and invasive tumor cells. To determine whether miR-29a inhibits the division and migration of HeLa cells, we performed wound healing and cell migration assays. The wound healing assay was conducted over 3 days, with measurements taken at 24 h intervals after creating a scratch in the wells. The results showed that the degree of wound healing in cells treated with scramble miRNA was 21.6%, 41.6%, and 71.1% at 24, 48, and 72 h, respectively. In contrast, the HeLa cells treated with miR-29a to artificially induce miR-29a overexpression exhibited wound healing of 11.9%, 24.4%, and 38.9% at the same time points (Figure 3a). This indicates that miR-29a reduces the growth and division of HPV type 18-infected HeLa cells to approximately half the level observed with scramble miRNA treatment.

Additionally, to assess whether miR-29a inhibits the invasive ability of HeLa cells, we conducted a cell migration assay. The results demonstrated that HeLa cells transfected with scramble miRNA migrated at an average of 1500 cells per randomly observed field of view, whereas miR-29a-treated HeLa cells exhibited significantly reduced migration, with approximately 350 cells per field of view (Figure 3b).

This represents a reduction of more than 80% in the invasive capacity of HeLa cells overexpressing miR-29a. As a result, these experimental findings suggest that miR-29a effectively inhibits cell proliferation and migration in HPV type 18-infected HeLa cells. Therefore, miR-29a may hold potential as a therapeutic agent for treating certain types of cervical cancer.

### 3.4. Validation of miR-29a’s Specific Binding to 3′-UTR of PIK3CA mRNA

**Figure 4:**
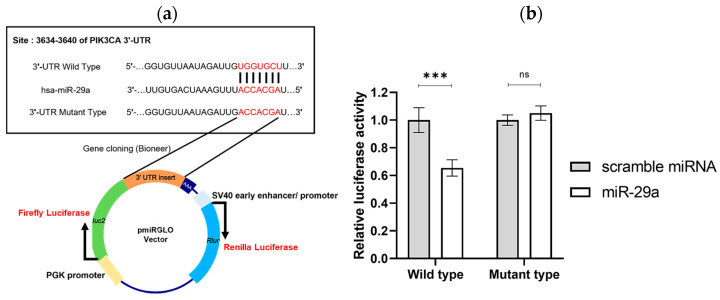
Specificity of miR-29a for PIK3CA 3′-UTR binding validated by luciferase activity. (**a**) Schematic representation of the pmiRGLO-3′UTR plasmids used for the luciferase assay, showing both the wild-type and mutant versions of the PIK3CA 3′-UTR. (**b**) Luminescence results from co-transfection of HeLa cells with either scramble miRNA or miR-29a and the pmiRGLO-3′UTR plasmids. The data indicate that miR-29a significantly reduces luciferase activity when the wild-type 3′-UTR is present, confirming specific binding to PIK3CA. Error bars represent the mean ± standard deviation (S.D.) of three independent experiments. Statistical significance is denoted as *** *p* < 0.001, ns means not significant.

Previous experiments demonstrated that miR-29a reduces PIK3CA expression at both RNA and protein levels and inhibits the proliferation and invasion of cervical cancer cells. To further confirm that miR-29a selectively binds to the 3′-UTR of PIK3CA and destabilizes it, thereby reducing gene expression, we created a specialized plasmid (Figure 4a and Figure S2). The pmiRGLO-3′UTR wild-type plasmid includes a 3′-UTR that can specifically bind to hsa-miR29a-3p (miR-29a) after the Firefly luciferase gene. In contrast, the pmiRGLO-3′UTR mutant plasmid contains a 3′-UTR that cannot bind to hsa-miR29a-3p, following the Renilla luciferase gene.

We then co-transfected each plasmid with either scramble miRNA or miR-29a and measured the luminescence. For the wild-type plasmid, the relative luciferase activity of the Firefly luciferase (with 3′-UTR) and Renilla luciferase was similar when transfected with scramble miRNA. However, upon miR-29a transfection, the Firefly luciferase expression significantly decreased (Figure 4b). In contrast, for the mutant plasmid, which has a 3′-UTR that does not bind miR-29a, the relative luciferase activity remained nearly identical for both scramble miRNA and miR-29a transfections. To elaborate on the significance of this experiment and the implications of the results, we utilized a plasmid called pmiRGLO with the 3′-UTR wild-type sequence of PIK3CA to investigate the interaction with hsa-miR-29a. When hsa-miR-29a binds to the 3′-UTR wild-type of pmiRGLO, the expression of Firefly luciferase, which is initially high, is reduced. This reduction is indicative of miR-29a’s ability to selectively bind to the 3′-UTR of PIK3CA, as the 3′-UTR sequence in pmiRGLO matches that of PIK3CA. The decrease in Firefly luciferase expression is quantitatively measured using a luminometer.

In contrast, when a mutant version of pmiRGLO, containing a 3′-UTR sequence that cannot bind to hsa-miR-29a, is used, the expression of Firefly luciferase remains unchanged. This lack of reduction indicates that miR-29a is unable to interact with the mutated sequence, resulting in consistent luminescence intensity. The Renilla luciferase expression, which serves as an internal control, also remains unchanged in all cases.

In conclusion, the dual-luciferase assay demonstrates that miR-29a selectively binds to the 3′-UTR of PIK3CA mRNA, leading to decreased gene expression. This finding underscores the role of miR-29a in the post-transcriptional regulation of PIK3CA and highlights its potential as a therapeutic target in cervical cancer.

### 3.5. Expression Levels of miR-29a and PIK3CA in Normal and Cervical Cancer Patients Analyzed by TCGA (The Cancer Genome Atlas Program)

**Figure 5:**
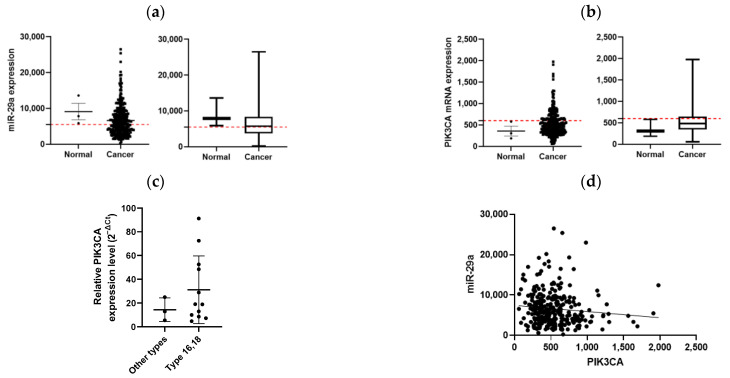
Analysis of miR-29a and PIK3CA expression levels in normal and cervical cancer samples. (**a**) Box plot comparing the relative expression levels of miR-29a between normal and cervical cancer patients. (**b**) Box plot comparing the relative expression levels of PIK3CA mRNA between normal and cervical cancer patients. (**c**) Analyses of tumor size, the relative expression levels of miR-29a, and PIK3CA in patients infected with high-risk HPV types (16 and 18). (**d**) Scatter plot showing the negative correlation between miR-29a and PIK3CA mRNA expression levels, based on the TCGA dataset. Error bars represent the standard deviation from three independent experiments.

To determine whether the expression levels of miR-29a differ between normal and cervical cancer patients, we analyzed the TCGA dataset. The results showed that the miR-29a expression levels in cervical cancer patients had a lower range and median compared to the normal samples, suggesting that miR-29a expression is reduced in the cancer samples (Figure 5a). Similarly, the PIK3CA mRNA expression levels displayed a higher range and median in cervical cancer patients, indicating that PIK3CA expression is elevated in the cancer samples (Figure 5b).

However, it is important to note that the TCGA dataset primarily consists of data from cancer patients, resulting in a relatively small number of normal samples. This discrepancy could limit the statistical power and generalizability of the comparisons between normal and cancer samples.

In addition, when comparing the tumor size from actual cervical cancer patients with the relative expression level of miR-29a, it was observed that a lower miR-29a expression was associated with larger tumor sizes. Furthermore, the PIK3CA expression levels varied significantly depending on the HPV infection type, with lower levels observed in patients infected with high-risk HPV types (16 and 18) compared to other HPV types (Figure 5c).

Therefore, the observation that miR-29a is lower in cancer samples while PIK3CA is higher supports the notion of a negative correlation between miR-29a and PIK3CA. This is consistent with the idea that miR-29a inhibits the expression of PIK3CA and supports the hypothesis that lower levels of miR-29a in cervical cancer lead to a higher expression of PIK3CA mRNA (Figure 5d).

### 3.6. Comparison of miR-29a and PIK3CA mRNA Levels in Cervical Cancer Patients

The histological clinical samples from patients with cervical cancer and non-cervical cancer were provided by Wonju Severance Christian Hospital. The expression levels of miR-29a and PIK3CA mRNA in these tissues were compared and analyzed using the FFPE method. The results showed that miR-29a expression was significantly higher in the cervical tissues of patients without cervical cancer compared to those with cervical cancer. Similarly, PIK3CA expression in the cervical biopsy tissues showed a significant difference between patients with and without cervical cancer.

**Figure 6:**
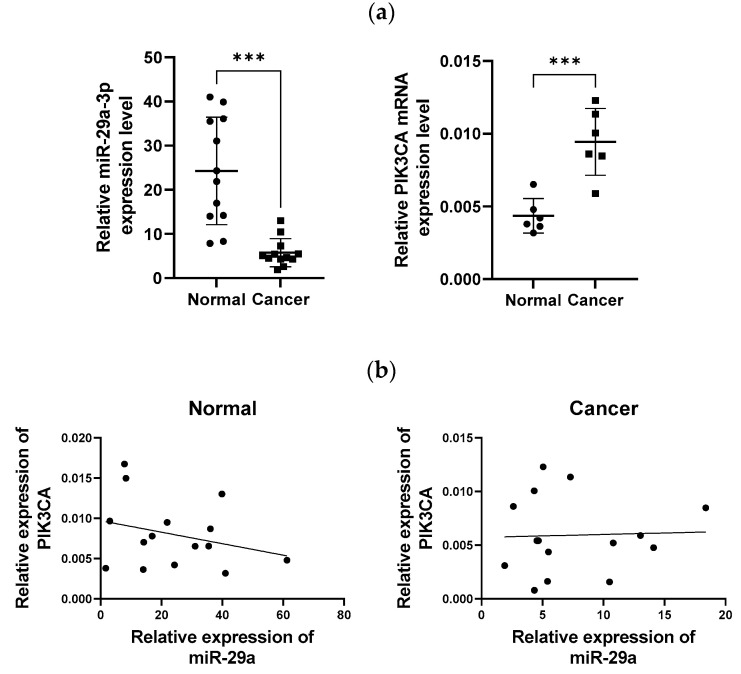
Analysis of miR-29a and PIK3CA mRNA expression levels in cervical cancer and non-cervical cancer tissues. (**a**) Box plot comparing the relative expression levels of miR-29a and PIK3CA mRNA in cervical tissue samples from patients with and without cervical cancer. The data demonstrate a significant difference in the expression levels of miR-29a and PIK3CA between the two groups. (**b**) Regression analysis plot showing the relationship between miR-29a and PIK3CA mRNA expression levels, indicating a potential correlation between these two markers. Statistical significance is denoted as *** *p* < 0.001.

These findings indicate that miR-29a expression is downregulated in the cervical biopsies of cervical cancer patients. Since PIK3CA is known to be overexpressed in cervical cancer, this study reaffirmed the significant difference in PIK3CA expression between normal and cancer tissues (Figure 6a). Furthermore, regression analysis revealed a relatively negative correlation between PIK3CA and miR-29a expression in the normal group, with PIK3CA expression decreasing as miR-29a increased. In contrast, the trend line of miR-29a and PIK3CA expression in the cervical cancer group was relatively flat. This suggests that the expression of miR-29a and PIK3CA is not significantly correlated in cervical cancer patients. However, considering the results of previous experiments, this indicates that the gene expression of miR-29a and PIK3CA is abnormally regulated in cervical cancer patients (Figure 6b). The information on the patients used in this study is attached to the supplementary data (Table S2). The supplementary data are the patient data used in the study and are minimized so that individuals cannot be identified.

## 4. Conclusions

In this study, we expanded on the known finding that miR-29a expression in vivo can regulate tumor development by exploring its relationship with cervical cancer. First, we examined the expression of PIK3CA, a gene related to the PIK3/AKT/mTOR signaling pathway, across various cervical cancer cell lines. We observed that the expression of PIK3CA varied depending on the cervical cancer profile, and this variation was mirrored by the miR-29a expression levels. When miR-29a was artificially overexpressed, we found that the RNA and protein levels of PIK3CA were significantly reduced, as demonstrated by RT-qPCR and Western blotting. This suggests that miR-29a functions as a gene silencer that can downregulate PIK3CA expression.

The reduced expression of PIK3CA, mediated by miR-29a, led to a decrease in the proliferation, division, erosion, and metastasis of cervical cancer cells, which was confirmed through wound healing and cell migration assays. To investigate whether miR-29a inhibits PIK3CA by affecting the PIK3/AKT/mTOR signaling pathway indirectly or by directly binding to the 3′-UTR of PIK3CA mRNA, we performed a dual-luciferase assay.

To validate the relationship between miR-29a and PIK3CA in clinical settings, we examined data from the TCGA dataset and found that cervical cancer patients generally exhibited a higher expression of PIK3CA and lower expression of miR-29a. Additionally, we observed the expression of miR-29a and PIK3CA in the biopsy specimens from cervical cancer patients and non-cervical cancer patients, finding that the miR-29a levels were lower and the PIK3CA levels were higher in cervical cancer patients compared to normal individuals. Although it remains uncertain whether the increased expression of PIK3CA is directly due to decreased miR-29a expression, this study is significant in revealing that miRNAs can act as gene therapeutic agents. It provides a new paradigm for diagnosing cervical cancer by examining the miR-29a and PIK3CA expression in actual patients.

Therefore, this study paves the way for further research on the therapeutic use of miRNAs, which naturally regulate gene expression, in treating cervical cancer. Future research should focus on the application of these therapeutic methods in clinical settings for cervical cancer patients.

## 5. Patents

HY, Lee, HR. Jeong., and Dasom Hwang. (2021). Nucleic acid fragment specifically binding to the 3′ untranslated region of the phosphatidylinositol 3-kinase catalytic subunit alpha (PIK3CA) gene and applications thereof (Korean Patent No. 10-2021-0192838), Korean intellectual property offices.

## Data Availability

Data are contained within the article.

## Supplementary Materials

The following supporting information can be downloaded from: https://www.mdpi.com/article/10.3390/cimb46110754/s1, Figure S1: Multiple alignments of the PIK3CA; Figure S2: A Predicted miR-29a binding site on 3′-UTR of PIK3CA mRNA. Table S1: Primers and probes used for analyses of mRNA expression in this study; Table S2: Clinical information on cervical cancer patients and normal controls.

## References

[1] Bray F., Laversanne M., Sung H., Ferlay J., Siegel R.L., Soerjomataram I., Jemal A. (2024). Global cancer statistics 2022: GLOBOCAN estimates of incidence and mortality worldwide for 36 cancers in 185 countries. CA Cancer J. Clin..

[2] Arbyn M., Weiderpass E., Bruni L., de Sanjosé S., Saraiya M., Ferlay J., Bray F. (2020). Estimates of incidence and mortality of cervical cancer in 2018: A worldwide analysis. Lancet Glob. Health.

[3] Simms K.T., Steinberg J., Caruana M., Smith M.A., Lew J.-B., Soerjomataram I., Castle P.E., Bray F., Canfell K. (2019). Impact of Scaled up Human Papillomavirus Vaccination and Cervical Screening and the Potential for Global Elimination of Cervical Cancer in 181 Countries, 2020–99: A Modelling Study. Obstet. Gynecol. Surv..

[4] Viveros-Carreño D., Fernandes A., Pareja R. (2023). Updates on cervical cancer prevention. Int. J. Gynecol. Cancer.

[5] Tsai H.T., Tsai Y.M., Yang S.F., Lee C.H., Lin L.Y., Lee S., Wu M.T. (2009). A notable accessory screening program for detection of cervical intraepithelial neoplasia. Pathol. Biol..

[6] Mattern J., Letendre I., Sibiude J., Pénager C., Jnifen A., Souare F., Ayel S., Nguyen T., Mandelbrot L. (2022). Diagnosis of advanced cervical cancer, missed opportunities?. BMC Women’s Health.

[7] Pfaendler K.S., Tewari K.S. (2016). Changing paradigms in the systemic treatment of advanced cervical cancer. Am. J. Obstet. Gynecol..

[8] Cooley J.J.P., Maguire F.B., Morris C.R., Parikh-Patel A., Abrahão R., Chen H.A., Keegan T.H.M. (2023). Cervical Cancer Stage at Diagnosis and Survival among Women ≥65 Years in California. Cancer Epidemiol. Biomark. Prev..

[9] Lee S.-W., Lee S.H., Kim J., Kim Y.-S., Yoon M.S., Jeong S., Kim J.H., Lee J., Eom K.-Y., Jeong B.K. (2017). Magnetic resonance imaging during definitive chemoradiotherapy can predict tumor recurrence and patient survival in locally advanced cervical cancer: A multi-institutional retrospective analysis of KROG 16-01. Gynecol. Oncol..

[10] Lim A., Sia S. (2012). Outcomes of Chemoradiotherapy in Cervical Cancer—The Western Australian Experience. Int. J. Radiat. Oncol. Biol. Phys..

[11] Ibrahim P.N., Zhang J., Zhang C., Bollag G., Desai M.C. (2013). Chapter Twenty-Six—Case History: Vemurafenib, a Potent, Selective, and First-in-Class Inhibitor of Mutant BRAF for the Treatment of Metastatic Melanoma. Annual Reports in Medicinal Chemistry.

[12] Bollag G., Tsai J., Zhang J., Zhang C., Ibrahim P., Nolop K., Hirth P. (2012). Vemurafenib: The first drug approved for BRAF-mutant cancer. Nat. Rev. Drug Discov..

[13] Ma Y.-Y., Wei S.-J., Lin Y.-C., Lung J.-C., Chang T.-C., Whang-Peng J., Liu J.M., Yang D.-M., Yang W.K., Shen C.-Y. (2000). PIK3CA as an oncogene in cervical cancer. Oncogene.

[14] Razia S., Nakayama K., Nakamura K., Ishibashi T., Ishikawa M., Minamoto T., Iida K., Otsuki Y., Nakayama S., Ishikawa N. (2019). Clinicopathological and biological analysis of PIK3CA mutation and amplification in cervical carcinomas. Exp. Ther. Med..

[15] Bertelsen B.I., Steine S.J., Sandvei R., Molven A., Laerum O.D. (2006). Molecular analysis of the PI3K-AKT pathway in uterine cervical neoplasia: Frequent PIK3CA amplification and AKT phosphorylation. Int. J. Cancer.

[16] Hudson K., Hancox U., Trigwell C., Dudley P., Hanson L., McEwen R., Jones A., Cumberbatch M., Polanska U., Ellston R. (2015). Abstract 2665: High dose intermittent scheduling of AZD8835, a novel potent and selective inhibitor of PI3Kα and PI3Kδ, identifies potential treatment strategies for PIK3CA-dependent cancers. Cancer Res..

[17] Jessen K., Kessler L., Kucharski J., Guo X., Staunton J., Janes M., Elia M., Banerjee U., Lan L., Wang S. (2011). Abstract A171: A potent and selective PI3K inhibitor, INK1117, targets human cancers harboring oncogenic PIK3CA mutations. Mol. Cancer Ther..

[18] Jackson R.J., Standart N. (2007). How Do MicroRNAs Regulate Gene Expression?. Sci. STKE.

[19] Sacco L.D., Masotti A. (2013). Recent Insights and Novel Bioinformatics Tools to Understand the Role of MicroRNAs Binding to 5′ Untranslated Region. Int. J. Mol. Sci..

[20] Filipowicz W., Jaskiewicz L., Kolb F.A., Pillai R.S. (2005). Post-transcriptional gene silencing by siRNAs and miRNAs. Curr. Opin. Struct. Biol..

[21] Lam J.K.W., Chow M.Y.T., Zhang Y., Leung S.W.S. (2015). siRNA Versus miRNA as Therapeutics for Gene Silencing. Mol. Ther. Nucleic Acids.

[22] Sun D., Melegari M., Sridhar S., Rogler C.E., Zhu L. (2006). Multi-miRNA hairpin method that improves gene knockdown efficiency and provides linked multi-gene knockdown. BioTechniques.

[23] Saito Y., Nakaoka T., Saito H. (2015). microRNA-34a as a Therapeutic Agent against Human Cancer. J. Clin. Med..

[24] Beg M.S., Hong D.S., Sachdev J.C., Brenner A.J., Borad M.J., Lim H.Y., Kim T.-Y., Becerra C., Park K., Bader A.G. (2016). First-in-human trial of microRNA cancer therapy with MRX34, a liposomal miR-34 mimic: Phase Ia expansion in patients with advanced solid tumors. J. Clin. Oncol..

[25] Hong D.S., Kang Y.-K., Borad M., Sachdev J., Ejadi S., Lim H.Y., Brenner A.J., Park K., Lee J.-L., Kim T.-Y. (2020). Phase 1 study of MRX34, a liposomal miR-34a mimic, in patients with advanced solid tumours. Br. J. Cancer.

[26] Wiggins J.F., Ruffino L., Kelnar K., Omotola M., Patrawala L., Brown D., Bader A.G. (2010). Development of a Lung Cancer Therapeutic Based on the Tumor Suppressor MicroRNA-34. Cancer Res..

[27] Pang R.T.K., Leung C.O.N., Ye T.-M., Liu W., Chiu P.C.N., Lam K.K.W., Lee K.-F., Yeung W.S.B. (2010). MicroRNA-34a suppresses invasion through downregulation of Notch1 and Jagged1 in cervical carcinoma and choriocarcinoma cells. Carcinogenesis.

[28] Schmitt M.J., Margue C., Behrmann I., Kreis S. (2013). MiRNA-29: A microRNA Family with Tumor-Suppressing and Immune-Modulating Properties. Curr. Mol. Med..

[29] Chen R., Zhang L. (2019). MiR-29a inhibits cell proliferation and migration by targeting the CDC42/PAK1 signaling pathway in cervical cancer. Anti-Cancer Drugs.

[30] Gong Y., Wan J.-H., Zou W., Lian G.-Y., Qin J.-L., Wang Q.-M. (2019). Mir-29A Inhibits Invasion and Metastasis of Cervical Cancer Via Modulating Methylation of Tumor Suppressor Socs1. Future Oncol..

