# Supplementary material for: miR-29a Downregulates PIK3CA Expression and Inhibits Cervical Cancer Cell Dynamics: A Comparative Clinical Analysis
Source: Curr Issues Mol Biol. 2024 Nov 8;46(11):12704–17. doi: 10.3390/cimb46110754 (PMC11592673; doi:10.3390/cimb46110754)
Supplement: Supplementary file 1 [file cimb-46-00754-s001.zip › cimb-3276344-supplementary.pdf]

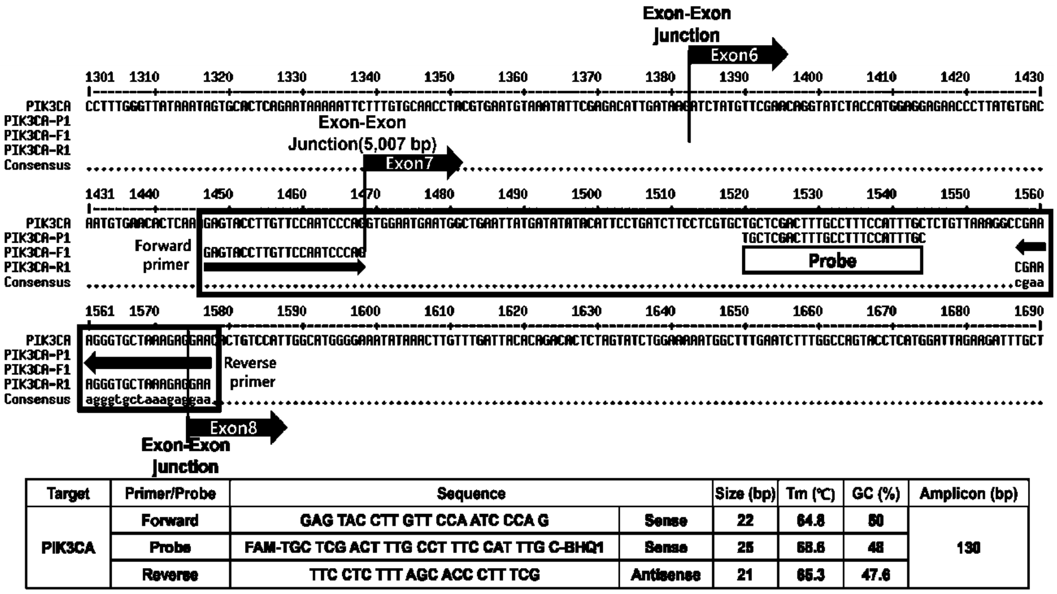

Figure S1. Multiple alignments of the PIK3CA primer set to detect mRNA of PIK3CA. For the primer design to detect mRNA of PIK3CA, the primer candidates for RT-qPCR of PIK3CA mRNA were selected by the sequence of Integrated DNA Technologies (IDT, Coralville, IA, USA)'s primer quest tool, oligo analyzer tool, basic local alignment search tool (BLAST by NCBI, Bethesda, MD, USA), and multiple alignments to detect only mRNA of PIK3CA.

Table S1. Primers and probes used for analyses of mRNA expression in this study

Target gene		Sequence (5'-3')	PCR-product (bp)	Annealing temperature (°C)	Cycle
GAPDH	F	CCATCTTCCAGGAGCGAGATCC	90	63	40
	R	ATGGTGGTGAAGACGCCAGTG			
	P	FAM-TCCACGACGTACTCAGCGCCAGCA-BHQ1			
PIK3CA	F	GAGTACCTTGTTCCAATCCCAG	130	63	40
	R	TTCCTCTTTAGCACCCTTTCG			
	P	FAM-TGCTCGACTTTGCCTTTCCATTTGC-BHQ1			

GAPDH, glyceraldehyde-3-phosphate dehydrogenase

PIK3CA, phosphatidylinositol 3 kinase catalytic subunit alpha

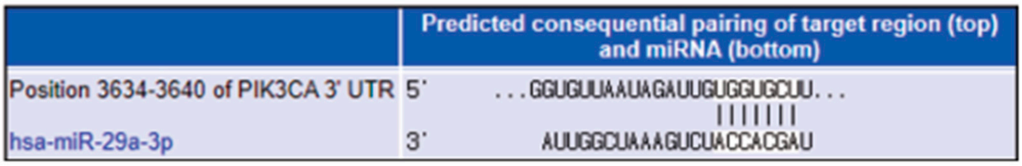

Figure S2. A predicted miR-29a binding site on 3′-UTR of PIK3CA mRNA. TargetScan software (http://www.targetscan.org/) was used to predict human miR-29a (hsa-miR-29a) target site on PIK3CA genes. The TargetScan algorithm searches the UTRs of mRNAs for segments with perfect complementarity to bases 2-8 of the miRNAs numbered from the 5' end and referred to as the miRNA seed region. When pre-miRNA duplex functions as mature miRNA by forming a complex with proteins, the 3' arm of pre-miRNA is mainly encased in proteins and the 5' arm is degraded, so the 3' arm of miR-29a (miR-29a-3p) was used here for the search.

Table S2. Clinical information on cervical cancer patients and normal controls

Features	Cervical cancer patients, N (%)	Normal controls, N (%)
**Age (years)**		
**<50**	6 (40)	7 (47)
**≥50**	9 (60)	8 (53)
**FIGO stage**		
**IA-IIA**	3 (20)	
**IIB-IVB**	10 (67)	
**Not recorded**	2 (13)	
**Histological type**		
**Invasive squamous cell carcinoma**	15 (100)	
**HPV type**		
**16, 18**	9 (60)	
**Other**	6 (40)	
**Survival**		
**Alive**	6 (40)	
**Died**	1 (7)	
**Not recorded**	8 (53)	
